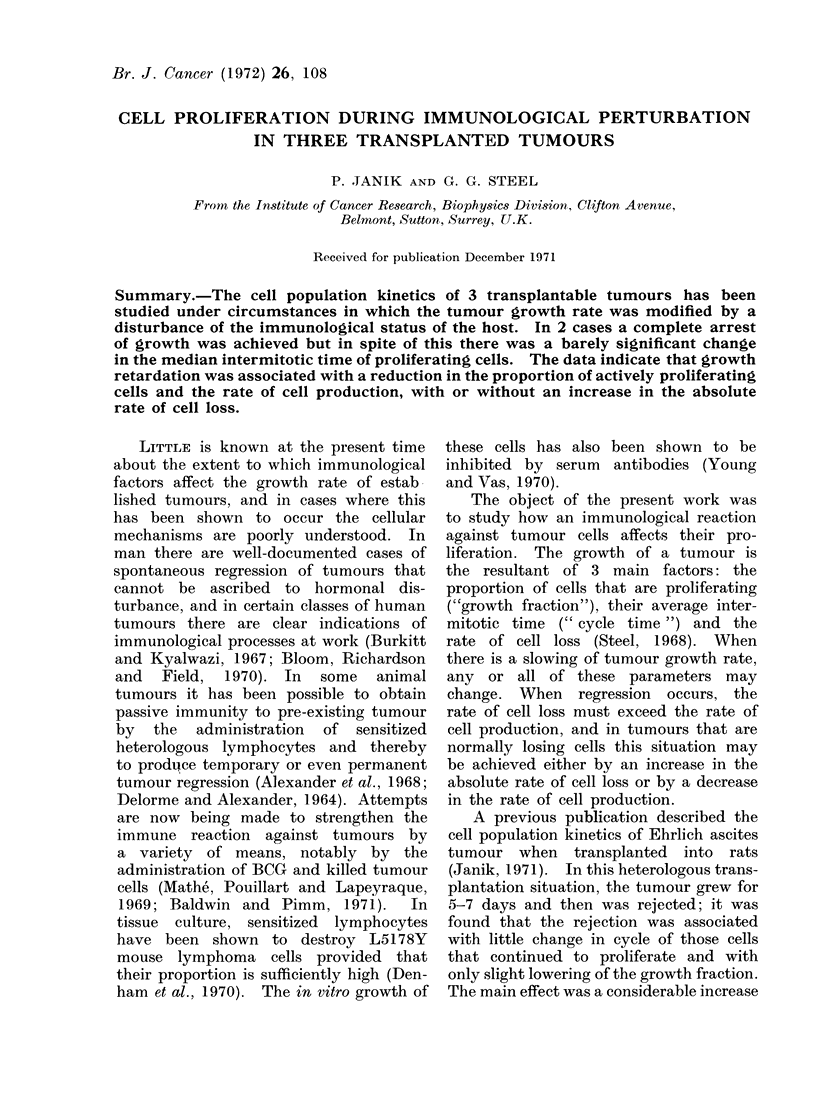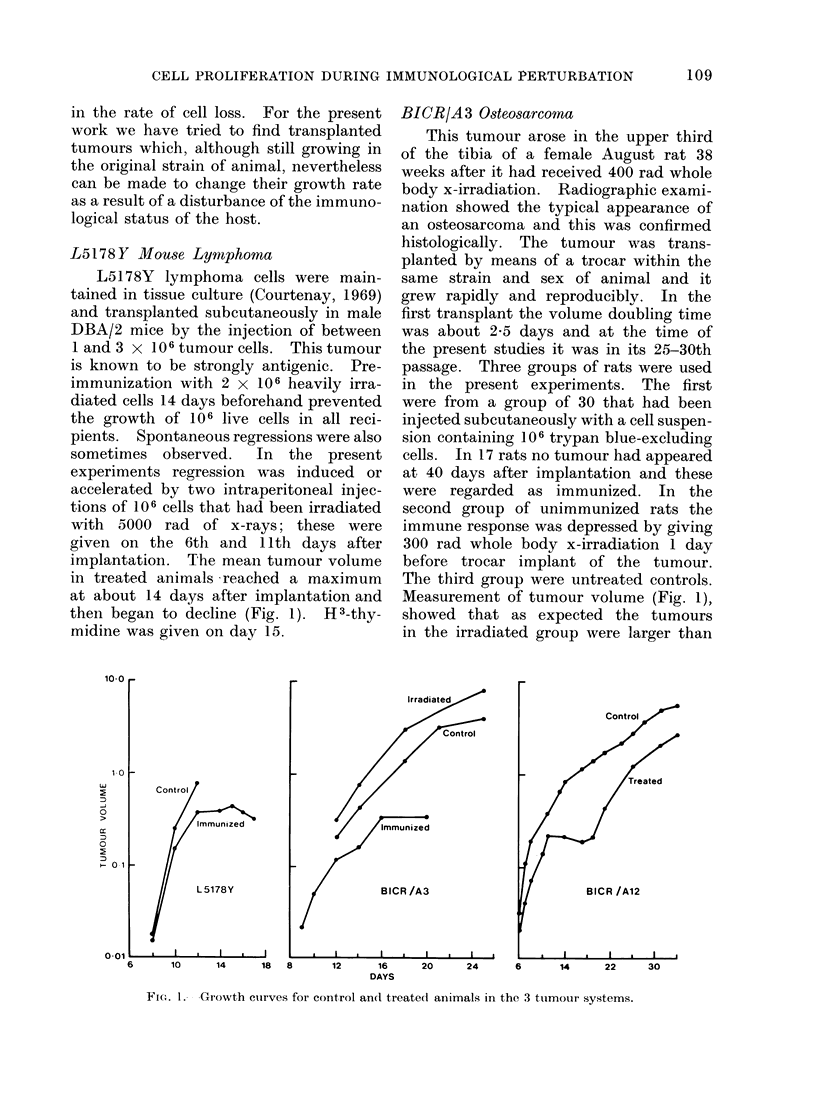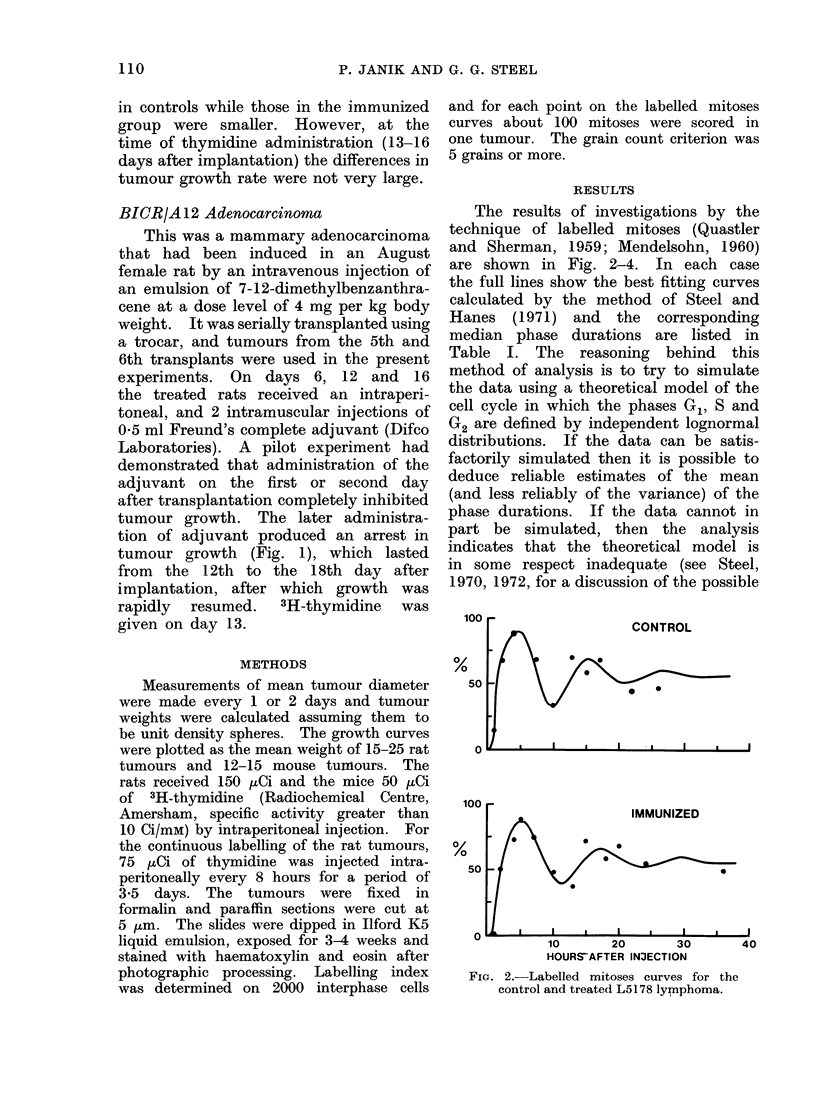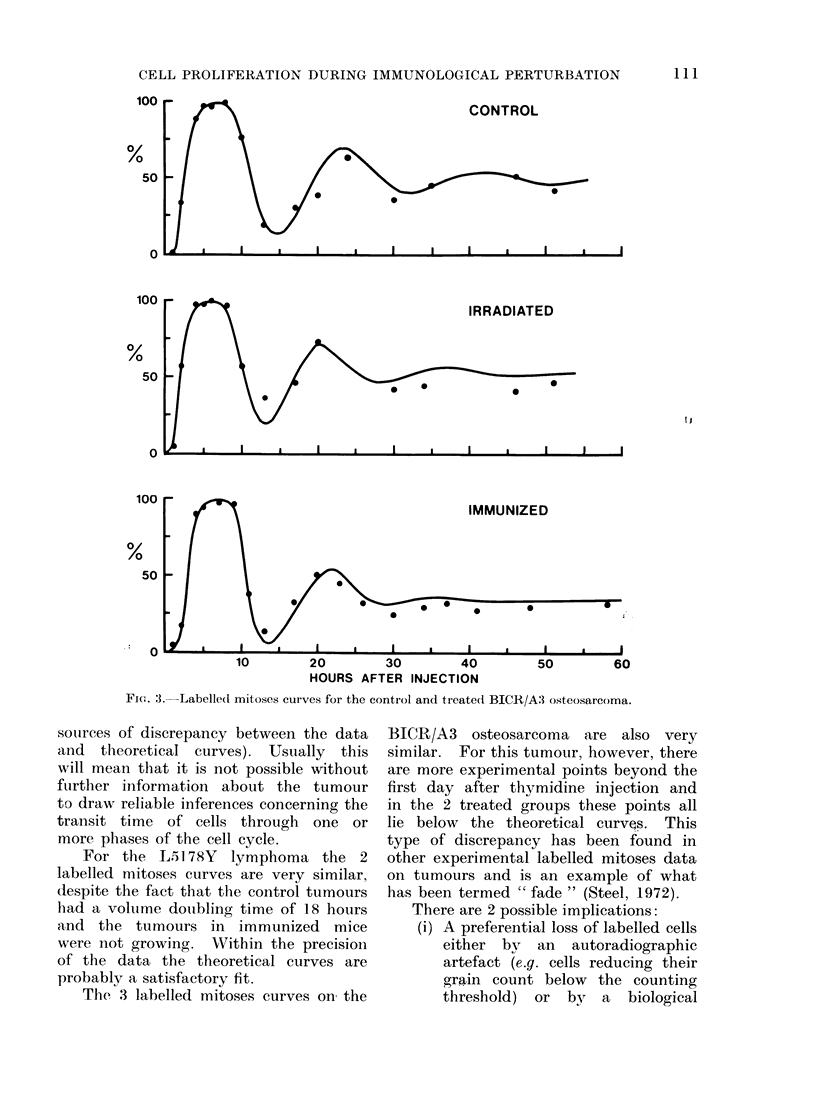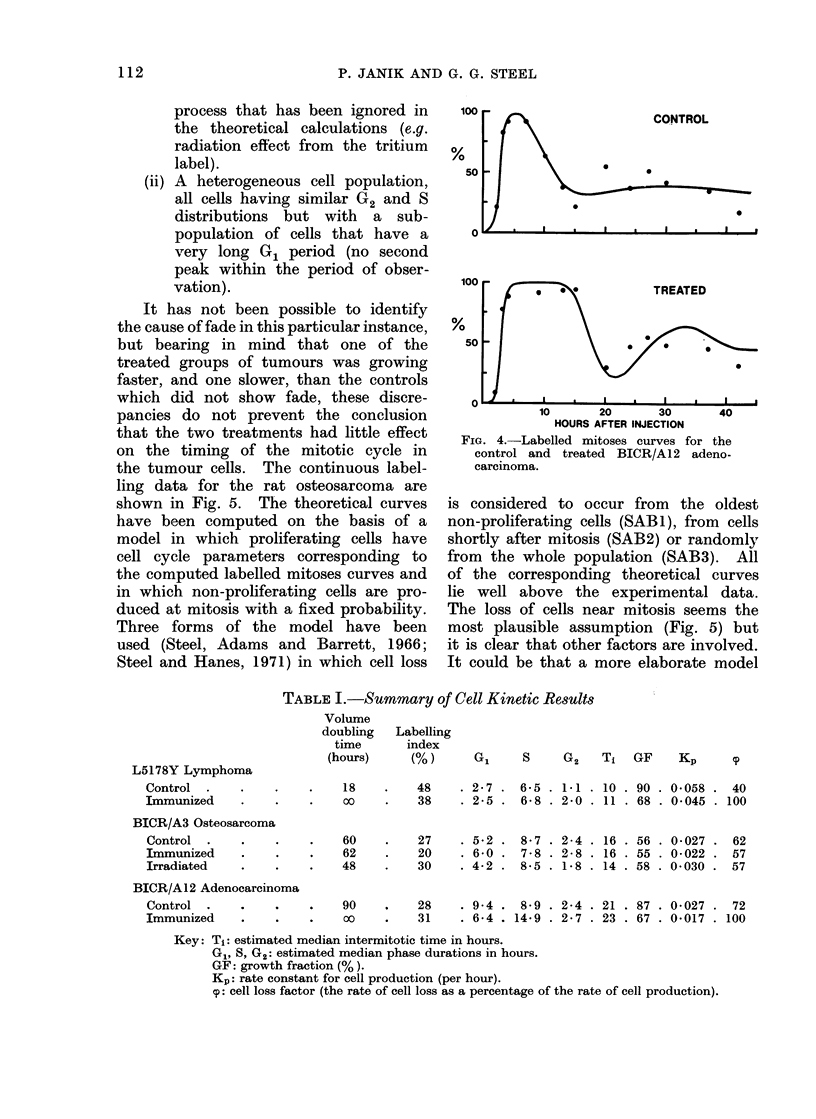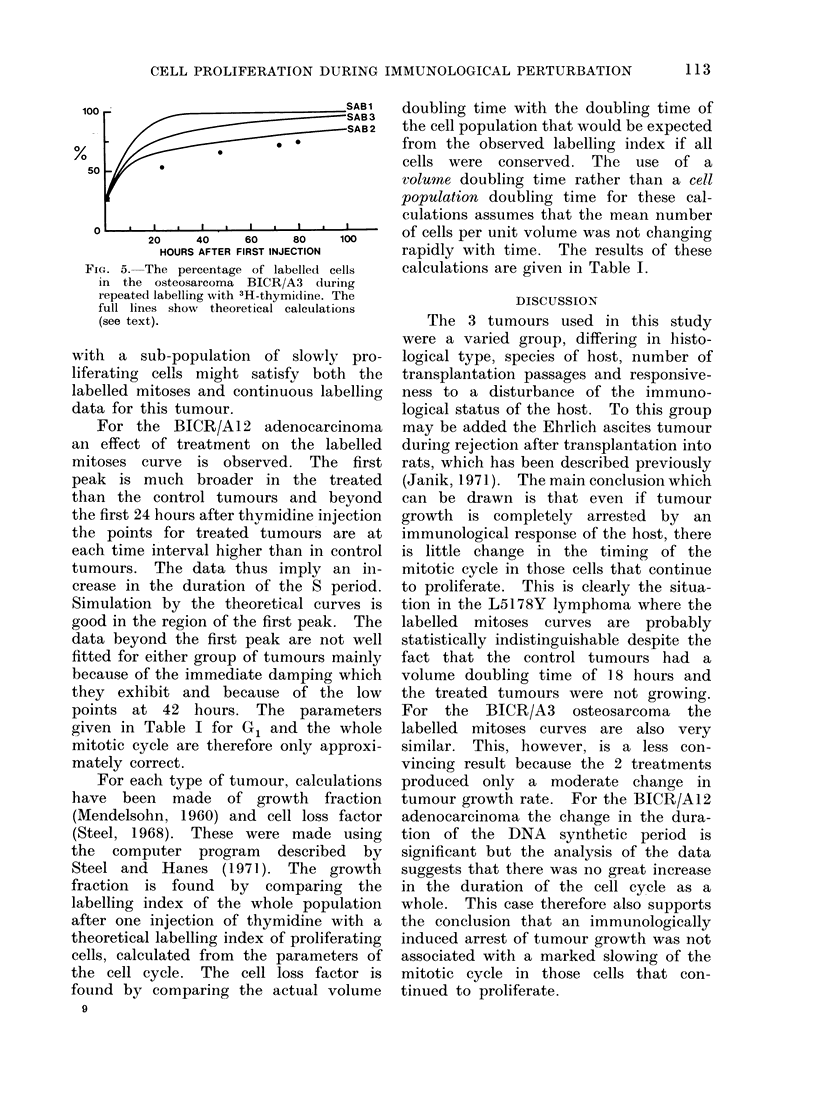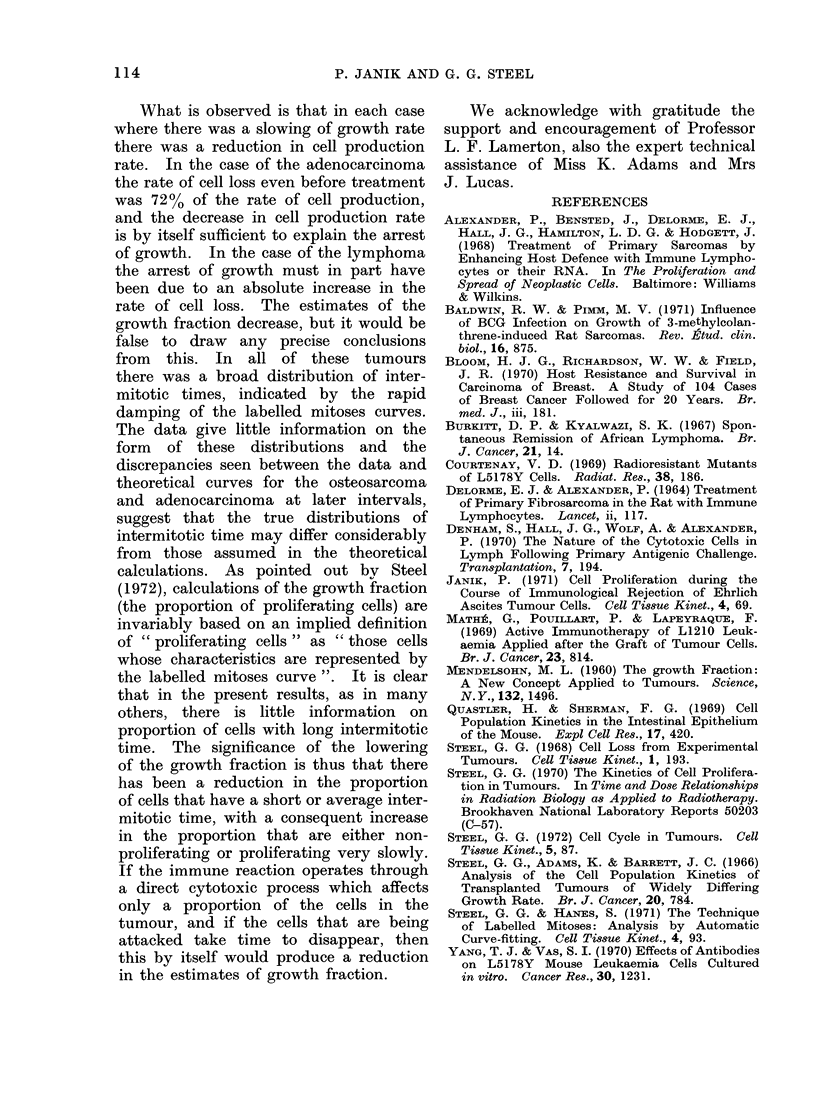# Cell Proliferation During Immunological Perturbation in Three Transplanted Tumours

**DOI:** 10.1038/bjc.1972.16

**Published:** 1972-04

**Authors:** P. Janik, G. G. Steel

## Abstract

The cell population kinetics of 3 transplantable tumours has been studied under circumstances in which the tumour growth rate was modified by a disturbance of the immunological status of the host. In 2 cases a complete arrest of growth was achieved but in spite of this there was a barely significant change in the median intermitotic time of proliferating cells. The data indicate that growth retardation was associated with a reduction in the proportion of actively proliferating cells and the rate of cell production, with or without an increase in the absolute rate of cell loss.


*Br. J. Cancer* (1972) **26**, 108

### CELL PROLIFERATION DURING IMMUNOLOGICAL PERTURBATION IN THREE TRANSPLANTED TUMOURS

P. JANIK AND G. G. STEEL

*From the Institute of Cancer Research, Biophysics Division, Clifton Avenue, Belmont, Sutton, Surrey, U.K.*

Received for publication December 1971

**Summary.—The cell population kinetics of 3 transplantable tumours has been studied under circumstances in which the tumour growth rate was modified by a disturbance of the immunological status of the host. In 2 cases a complete arrest of growth was achieved but in spite of this there was a barely significant change in the median intermitotic time of proliferating cells. The data indicate that growth retardation was associated with a reduction in the proportion of actively proliferating cells and the rate of cell production, with or without an increase in the absolute rate of cell loss.**

LITTLE is known at the present time about the extent to which immunological factors affect the growth rate of established tumours, and in cases where this has been shown to occur the cellular mechanisms are poorly understood. In man there are well-documented cases of spontaneous regression of tumours that cannot be ascribed to hormonal disturbance, and in certain classes of human tumours there are clear indications of immunological processes at work (Burkitt and Kyalwazi, 1967; Bloom, Richardson and Field, 1970). In some animal tumours it has been possible to obtain passive immunity to pre-existing tumour by the administration of sensitized heterologous lymphocytes and thereby to produce temporary or even permanent tumour regression (Alexander *et al.*, 1968; Delorme and Alexander, 1964). Attempts are now being made to strengthen the immune reaction against tumours by a variety of means, notably by the administration of BCG and killed tumour cells (Mathé, Pouillart and Lapeyraque, 1969; Baldwin and Pimm, 1971). In tissue culture, sensitized lymphocytes have been shown to destroy L5178Y mouse lymphoma cells provided that their proportion is sufficiently high (Denham *et al.*, 1970). The *in vitro* growth of these cells has also been shown to be inhibited by serum antibodies (Young and Vas, 1970).

The object of the present work was to study how an immunological reaction against tumour cells affects their proliferation. The growth of a tumour is the resultant of 3 main factors: the proportion of cells that are proliferating ("growth fraction"), their average intermitotic time (" cycle time ") and the rate of cell loss (Steel, 1968). When there is a slowing of tumour growth rate, any or all of these parameters may change. When regression occurs, the rate of cell loss must exceed the rate of cell production, and in tumours that are normally losing cells this situation may be achieved either by an increase in the absolute rate of cell loss or by a decrease in the rate of cell production.

A previous publication described the cell population kinetics of Ehrlich ascites tumour when transplanted into rats (Janik, 1971). In this heterologous transplantation situation, the tumour grew for 5–7 days and then was rejected; it was found that the rejection was associated with little change in cycle of those cells that continued to proliferate and with only slight lowering of the growth fraction. The main effect was a considerable increase in the rate of cell loss. For the present work we have tried to find transplanted tumours which, although still growing in the original strain of animal, nevertheless can be made to change their growth rate as a result of a disturbance of the immunological status of the host.

*L5178Y Mouse Lymphoma*

L5178Y lymphoma cells were maintained in tissue culture (Courtenay, 1969) and transplanted subcutaneously in male DBA/2 mice by the injection of between 1 and 3 $\times$ $10^6$ tumour cells. This tumour is known to be strongly antigenic. Preimmunization with 2 $\times$ $10^6$ heavily irradiated cells 14 days beforehand prevented the growth of $10^6$ live cells in all recipients. Spontaneous regressions were also sometimes observed. In the present experiments regression was induced or accelerated by two intraperitoneal injections of $10^6$ cells that had been irradiated with 5000 rad of x-rays; these were given on the 6th and 11th days after implantation. The mean tumour volume in treated animals reached a maximum at about 14 days after implantation and then began to decline (Fig. 1). $H^3$-thymidine was given on day 15.

*BICR/A3 Osteosarcoma*

This tumour arose in the upper third of the tibia of a female August rat 38 weeks after it had received 400 rad whole body x-irradiation. Radiographic examination showed the typical appearance of an osteosarcoma and this was confirmed histologically. The tumour was transplanted by means of a trocar within the same strain and sex of animal and it grew rapidly and reproducibly. In the first transplant the volume doubling time was about 2·5 days and at the time of the present studies it was in its 25–30th passage. Three groups of rats were used in the present experiments. The first were from a group of 30 that had been injected subcutaneously with a cell suspension containing $10^6$ trypan blue-excluding cells. In 17 rats no tumour had appeared at 40 days after implantation and these were regarded as immunized. In the second group of unimmunized rats the immune response was depressed by giving 300 rad whole body x-irradiation 1 day before trocar implant of the tumour. The third group were untreated controls. Measurement of tumour volume (Fig. 1), showed that as expected the tumours in the irradiated group were larger than

FIG. 1.—Growth curves for control and treated animals in the 3 tumour systems.

in controls while those in the immunized group were smaller. However, at the time of thymidine administration (13–16 days after implantation) the differences in tumour growth rate were not very large.

*BICR/A12 Adenocarcinoma*

This was a mammary adenocarcinoma that had been induced in an August female rat by an intravenous injection of an emulsion of 7-12-dimethylbenzanthracene at a dose level of 4 mg per kg body weight. It was serially transplanted using a trocar, and tumours from the 5th and 6th transplants were used in the present experiments. On days 6, 12 and 16 the treated rats received an intraperitoneal, and 2 intramuscular injections of 0·5 ml Freund's complete adjuvant (Difco Laboratories). A pilot experiment had demonstrated that administration of the adjuvant on the first or second day after transplantation completely inhibited tumour growth. The later administration of adjuvant produced an arrest in tumour growth (Fig. 1), which lasted from the 12th to the 18th day after implantation, after which growth was rapidly resumed. $^{3}$H-thymidine was given on day 13.

### METHODS

Measurements of mean tumour diameter were made every 1 or 2 days and tumour weights were calculated assuming them to be unit density spheres. The growth curves were plotted as the mean weight of 15–25 rat tumours and 12–15 mouse tumours. The rats received 150 $\mu$Ci and the mice 50 $\mu$Ci of $^{3}$H-thymidine (Radiochemical Centre, Amersham, specific activity greater than 10 Ci/mM) by intraperitoneal injection. For the continuous labelling of the rat tumours, 75 $\mu$Ci of thymidine was injected intraperitoneally every 8 hours for a period of 3·5 days. The tumours were fixed in formalin and paraffin sections were cut at 5 $\mu$m. The slides were dipped in Ilford K5 liquid emulsion, exposed for 3–4 weeks and stained with haematoxylin and eosin after photographic processing. Labelling index was determined on 2000 interphase cells and for each point on the labelled mitoses curves about 100 mitoses were scored in one tumour. The grain count criterion was 5 grains or more.

### RESULTS

The results of investigations by the technique of labelled mitoses (Quastler and Sherman, 1959; Mendelsohn, 1960) are shown in Fig. 2–4. In each case the full lines show the best fitting curves calculated by the method of Steel and Hanes (1971) and the corresponding median phase durations are listed in Table I. The reasoning behind this method of analysis is to try to simulate the data using a theoretical model of the cell cycle in which the phases $G_1$, S and $G_2$ are defined by independent lognormal distributions. If the data can be satisfactorily simulated then it is possible to deduce reliable estimates of the mean (and less reliably of the variance) of the phase durations. If the data cannot in part be simulated, then the analysis indicates that the theoretical model is in some respect inadequate (see Steel, 1970, 1972, for a discussion of the possible

FIG. 2.—Labelled mitoses curves for the control and treated L5178 lymphoma.

FIG. 3.—Labelled mitoses curves for the control and treated BICR/A3 osteosarcoma.

sources of discrepancy between the data and theoretical curves). Usually this will mean that it is not possible without further information about the tumour to draw reliable inferences concerning the transit time of cells through one or more phases of the cell cycle.

For the L5178Y lymphoma the 2 labelled mitoses curves are very similar, despite the fact that the control tumours had a volume doubling time of 18 hours and the tumours in immunized mice were not growing. Within the precision of the data the theoretical curves are probably a satisfactory fit.

The 3 labelled mitoses curves on the BICR/A3 osteosarcoma are also very similar. For this tumour, however, there are more experimental points beyond the first day after thymidine injection and in the 2 treated groups these points all lie below the theoretical curves. This type of discrepancy has been found in other experimental labelled mitoses data on tumours and is an example of what has been termed " fade " (Steel, 1972).

There are 2 possible implications:

(i) A preferential loss of labelled cells either by an autoradiographic artefact (*e.g.* cells reducing their grain count below the counting threshold) or by a biological process that has been ignored in the theoretical calculations (*e.g.* radiation effect from the tritium label).

(ii) A heterogeneous cell population, all cells having similar $G_2$ and S distributions but with a sub-population of cells that have a very long $G_1$ period (no second peak within the period of observation).

It has not been possible to identify the cause of fade in this particular instance, but bearing in mind that one of the treated groups of tumours was growing faster, and one slower, than the controls which did not show fade, these discrepancies do not prevent the conclusion that the two treatments had little effect on the timing of the mitotic cycle in the tumour cells. The continuous labelling data for the rat osteosarcoma are shown in Fig. 5. The theoretical curves have been computed on the basis of a model in which proliferating cells have cell cycle parameters corresponding to the computed labelled mitoses curves and in which non-proliferating cells are produced at mitosis with a fixed probability. Three forms of the model have been used (Steel, Adams and Barrett, 1966; Steel and Hanes, 1971) in which cell loss is considered to occur from the oldest non-proliferating cells (SAB1), from cells shortly after mitosis (SAB2) or randomly from the whole population (SAB3). All of the corresponding theoretical curves lie well above the experimental data. The loss of cells near mitosis seems the most plausible assumption (Fig. 5) but it is clear that other factors are involved. It could be that a more elaborate model

FIG. 4.—Labelled mitoses curves for the control and treated BICR/A12 adenocarcinoma.

TABLE I.—*Summary of Cell Kinetic Results*

| | Volume doubling time (hours) | Labelling index (%) | $G_1$ | S | $G_2$ | $T_i$ | GF | $K_p$ | φ |
|---|---|---|---|---|---|---|---|---|---|
| L5178Y Lymphoma | | | | | | | | | |
| Control | 18 | 48 | 2·7 | 6·5 | 1·1 | 10 | 90 | 0·058 | 40 |
| Immunized | ∞ | 38 | 2·5 | 6·8 | 2·0 | 11 | 68 | 0·045 | 100 |
| BICR/A3 Osteosarcoma | | | | | | | | | |
| Control | 60 | 27 | 5·2 | 8·7 | 2·4 | 16 | 56 | 0·027 | 62 |
| Immunized | 62 | 20 | 6·0 | 7·8 | 2·8 | 16 | 55 | 0·022 | 57 |
| Irradiated | 48 | 30 | 4·2 | 8·5 | 1·8 | 14 | 58 | 0·030 | 57 |
| BICR/A12 Adenocarcinoma | | | | | | | | | |
| Control | 90 | 28 | 9·4 | 8·9 | 2·4 | 21 | 87 | 0·027 | 72 |
| Immunized | ∞ | 31 | 6·4 | 14·9 | 2·7 | 23 | 67 | 0·017 | 100 |

Key: $T_i$: estimated median intermitotic time in hours.
$G_1$, S, $G_2$: estimated median phase durations in hours.
GF: growth fraction (%).
$K_p$: rate constant for cell production (per hour).
φ: cell loss factor (the rate of cell loss as a percentage of the rate of cell production).

FIG. 5.—The percentage of labelled cells in the osteosarcoma BICR/A3 during repeated labelling with $^3$H-thymidine. The full lines show theoretical calculations (see text).

with a sub-population of slowly proliferating cells might satisfy both the labelled mitoses and continuous labelling data for this tumour.

For the BICR/A12 adenocarcinoma an effect of treatment on the labelled mitoses curve is observed. The first peak is much broader in the treated than the control tumours and beyond the first 24 hours after thymidine injection the points for treated tumours are at each time interval higher than in control tumours. The data thus imply an increase in the duration of the S period. Simulation by the theoretical curves is good in the region of the first peak. The data beyond the first peak are not well fitted for either group of tumours mainly because of the immediate damping which they exhibit and because of the low points at 42 hours. The parameters given in Table I for $G_1$ and the whole mitotic cycle are therefore only approximately correct.

For each type of tumour, calculations have been made of growth fraction (Mendelsohn, 1960) and cell loss factor (Steel, 1968). These were made using the computer program described by Steel and Hanes (1971). The growth fraction is found by comparing the labelling index of the whole population after one injection of thymidine with a theoretical labelling index of proliferating cells, calculated from the parameters of the cell cycle. The cell loss factor is found by comparing the actual volume doubling time with the doubling time of the cell population that would be expected from the observed labelling index if all cells were conserved. The use of a *volume* doubling time rather than a *cell population* doubling time for these calculations assumes that the mean number of cells per unit volume was not changing rapidly with time. The results of these calculations are given in Table I.

### DISCUSSION

The 3 tumours used in this study were a varied group, differing in histological type, species of host, number of transplantation passages and responsiveness to a disturbance of the immunological status of the host. To this group may be added the Ehrlich ascites tumour during rejection after transplantation into rats, which has been described previously (Janik, 1971). The main conclusion which can be drawn is that even if tumour growth is completely arrested by an immunological response of the host, there is little change in the timing of the mitotic cycle in those cells that continue to proliferate. This is clearly the situation in the L5178Y lymphoma where the labelled mitoses curves are probably statistically indistinguishable despite the fact that the control tumours had a volume doubling time of 18 hours and the treated tumours were not growing. For the BICR/A3 osteosarcoma the labelled mitoses curves are also very similar. This, however, is a less convincing result because the 2 treatments produced only a moderate change in tumour growth rate. For the BICR/A12 adenocarcinoma the change in the duration of the DNA synthetic period is significant but the analysis of the data suggests that there was no great increase in the duration of the cell cycle as a whole. This case therefore also supports the conclusion that an immunologically induced arrest of tumour growth was not associated with a marked slowing of the mitotic cycle in those cells that continued to proliferate.

What is observed is that in each case where there was a slowing of growth rate there was a reduction in cell production rate. In the case of the adenocarcinoma the rate of cell loss even before treatment was 72% of the rate of cell production, and the decrease in cell production rate is by itself sufficient to explain the arrest of growth. In the case of the lymphoma the arrest of growth must in part have been due to an absolute increase in the rate of cell loss. The estimates of the growth fraction decrease, but it would be false to draw any precise conclusions from this. In all of these tumours there was a broad distribution of intermitotic times, indicated by the rapid damping of the labelled mitoses curves. The data give little information on the form of these distributions and the discrepancies seen between the data and theoretical curves for the osteosarcoma and adenocarcinoma at later intervals, suggest that the true distributions of intermitotic time may differ considerably from those assumed in the theoretical calculations. As pointed out by Steel (1972), calculations of the growth fraction (the proportion of proliferating cells) are invariably based on an implied definition of " proliferating cells " as " those cells whose characteristics are represented by the labelled mitoses curve ". It is clear that in the present results, as in many others, there is little information on proportion of cells with long intermitotic time. The significance of the lowering of the growth fraction is thus that there has been a reduction in the proportion of cells that have a short or average intermitotic time, with a consequent increase in the proportion that are either non-proliferating or proliferating very slowly. If the immune reaction operates through a direct cytotoxic process which affects only a proportion of the cells in the tumour, and if the cells that are being attacked take time to disappear, then this by itself would produce a reduction in the estimates of growth fraction.

We acknowledge with gratitude the support and encouragement of Professor L. F. Lamerton, also the expert technical assistance of Miss K. Adams and Mrs J. Lucas.

### REFERENCES

Alexander, P., Bensted, J., Delorme, E. J., Hall, J. G., Hamilton, L. D. G. & Hodgett, J. (1968) Treatment of Primary Sarcomas by Enhancing Host Defence with Immune Lymphocytes or their RNA. In *The Proliferation and Spread of Neoplastic Cells*. Baltimore: Williams & Wilkins.

Baldwin, R. W. & Pimm, M. V. (1971) Influence of BCG Infection on Growth of 3-methylcolanthrene-induced Rat Sarcomas. *Rev. Étud. clin. biol.*, **16**, 875.

Bloom, H. J. G., Richardson, W. W. & Field, J. R. (1970) Host Resistance and Survival in Carcinoma of Breast. A Study of 104 Cases of Breast Cancer Followed for 20 Years. *Br. med. J.*, iii, 181.

Burkitt, D. P. & Kyalwazi, S. K. (1967) Spontaneous Remission of African Lymphoma. *Br. J. Cancer*, **21**, 14.

Courtenay, V. D. (1969) Radioresistant Mutants of L5178Y Cells. *Radiat. Res.*, **38**, 186.

Delorme, E. J. & Alexander, P. (1964) Treatment of Primary Fibrosarcoma in the Rat with Immune Lymphocytes. *Lancet*, ii, 117.

Denham, S., Hall, J. G., Wolf, A. & Alexander, P. (1970) The Nature of the Cytotoxic Cells in Lymph Following Primary Antigenic Challenge. *Transplantation*, **7**, 194.

Janik, P. (1971) Cell Proliferation during the Course of Immunological Rejection of Ehrlich Ascites Tumour Cells. *Cell Tissue Kinet.*, **4**, 69.

Mathé, G., Pouillart, P. & Lapeyraque, F. (1969) Active Immunotherapy of L1210 Leukaemia Applied after the Graft of Tumour Cells. *Br. J. Cancer*, **23**, 814.

Mendelsohn, M. L. (1960) The growth Fraction: A New Concept Applied to Tumours. *Science, N.Y.*, **132**, 1496.

Quastler, H. & Sherman, F. G. (1969) Cell Population Kinetics in the Intestinal Epithelium of the Mouse. *Expl Cell Res.*, **17**, 420.

Steel, G. G. (1968) Cell Loss from Experimental Tumours. *Cell Tissue Kinet.*, **1**, 193.

Steel, G. G. (1970) The Kinetics of Cell Proliferation in Tumours. In *Time and Dose Relationships in Radiation Biology as Applied to Radiotherapy*. Brookhaven National Laboratory Reports 50203 (C–57).

Steel, G. G. (1972) Cell Cycle in Tumours. *Cell Tissue Kinet.*, **5**, 87.

Steel, G. G., Adams, K. & Barrett, J. C. (1966) Analysis of the Cell Population Kinetics of Transplanted Tumours of Widely Differing Growth Rate. *Br. J. Cancer*, **20**, 784.

Steel, G. G. & Hanes, S. (1971) The Technique of Labelled Mitoses: Analysis by Automatic Curve-fitting. *Cell Tissue Kinet.*, **4**, 93.

Yang, T. J. & Vas, S. I. (1970) Effects of Antibodies on L5178Y Mouse Leukaemia Cells Cultured *in vitro*. *Cancer Res.*, **30**, 1231.